# Factors Associated With Intraoral Skin Graft Take Following Oral Cavity Surgery

**DOI:** 10.7759/cureus.109287

**Published:** 2026-05-20

**Authors:** Troy Weinstein, Claire Gleadhill, Luis Helfer, Vicki Liu, Shethal Bearelly

**Affiliations:** 1 Department of Otolaryngology - Head and Neck Surgery, University of Arizona College of Medicine - Tucson, Tucson, USA; 2 Department of Surgery, University of Arizona College of Medicine - Tucson, Tucson, USA

**Keywords:** functional outcomes, head and neck surgery, oral cavity cancer, oral cavity reconstruction, skin graft take, split-thickness skin graft, tie-over bolster, tongue tethering

## Abstract

Objectives: To evaluate factors associated with graft success, tongue tethering, and dysphagia following intraoral skin grafting.

Methods: In this retrospective case series, we evaluated adult patients from 2018 to 2024 who underwent intraoral split-thickness skin graft (STSG) for oral cavity defects. Our primary outcome was complete skin graft take, with a secondary outcome of tongue tethering and dysphagia. Fisher's exact test was used for the comparison of categorical variables, and Mann-Whitney U tests were used for continuous variables.

Results: Thirty-nine patients were included. The mean age (SD) was 63.9 (13.2), and 59% were male. Mean follow-up (SD) was 1.66 years (1.54). The average defect area (SD) was 11.13 cm^2^ (4.24). Seven (17.9%) had successful skin graft take. Bolster placement (p = 0.059) and pie-crusting (p = 0.057) both trended toward association with successful graft take. Smoking history, defect area, T-stage, tumor location, postoperative antibiotics, radiation, and chemotherapy were not significantly associated with skin graft success. Tongue tethering occurred in seven patients (17.9%), and dysphagia occurred in six patients (15.4%). None of the above-reported variables were significantly associated with tongue tethering, but radiation (p = 0.011) and chemotherapy (p = 0.006) were both significantly associated with dysphagia.

Conclusions: Bolster placement and pie-crusting trended toward improved graft survival, whereas demographic and clinical factors, including age, sex, smoking status, comorbidities, tumor stage, and tumor location, were not predictive. Dysphagia was significantly associated with radiation and/or chemotherapy. Larger, prospective studies are needed to better define predictors of STSG success.

## Introduction

Oral cavity cancers are among the most common malignancies of the head and neck region, representing approximately 40% of head and neck squamous cell carcinoma (SCC) cases [1]. Surgical excision is typically the first-line treatment, often followed by radiation or chemotherapy [2,3]. However, these resections may result in large intraoral defects that impair speech, swallowing, and facial appearance, significantly impacting function and quality of life [4-6]. Small wounds may heal by primary closure, in which wound edges are directly approximated, or by secondary intention through granulation, contraction, and epithelialization. However, larger defects require reconstruction, for which intraoral split-thickness skin grafts (STSG) are commonly used [5,7]. Compared to bulkier reconstructive options, such as myocutaneous grafts, STSGs preserve tongue mobility and swallowing and may also reduce the risk of tongue tethering [5,8]. Although alternative graft materials, such as dermal matrices and tissue-engineered mucosa, have been explored, autologous STSGs remain the preferred option due to their functional outcomes, ease of use, and low donor site morbidity [9].

Despite the variety of grafting strategies, resurfacing intraoral defects presents unique challenges that hinder graft integration and survival. Factors such as irregular wound beds, salivary secretions, and continuous mechanical stress from speech and mastication complicate graft take [4,9]. Prior studies have explored various operative techniques to improve intraoral graft outcomes, such as securing with silicone and tongue sandwiching using buttons combined with a tie-over [10,11]. However, despite these advancements, limitations in graft fixation and mucosalization in the oral cavity persist, especially in large or mobile defects [12,13]. Few studies have examined clinical and operative predictors of successful STSG take. A clearer understanding of these factors could help optimize graft outcomes and improve patient quality of life.

This single-center retrospective study evaluates clinical outcomes in patients who underwent STSG for the resection of oral cavity malignancies. The primary aim was to identify clinical and operative factors associated with skin graft success. Secondary aims included assessing tongue tethering and dysphagia, with the broader goal of informing surgical decision-making in this challenging anatomical region.

## Materials and methods

This study is a retrospective case series of 39 adult patients presenting to the University of Arizona head and neck surgical oncology team between 2018 and 2024 for excision of oral cavity defects. All patients underwent excision of the intraoral defect and had an intraoral split-thickness skin graft (STSG) placed. Diagnosis included SCC, high-grade dysplasia, and hyperkeratosis. One patient had a high-grade undifferentiated sarcoma, not otherwise specified (NOS). Patients were included if the graft was placed on the floor of mouth (FOM), tongue, or buccal mucosa. Patients who had a free flap reconstruction were excluded from the study. All patients had subsequent follow-up in the clinic.

The primary outcome was skin graft take, assessed by intraoral physical examination at the patient's most recent documented clinic visit. Graft success was defined as a fully intact, viable graft. Grafts were classified as failed if the examiner noted graft loss or sloughing, or if graft presence was not mentioned.

Secondary outcomes included tongue tethering and dysphagia. Tongue tethering, defined as restricted mobility of the residual tongue due to postoperative scarring, was assessed at the most recent clinic visit based on standard intraoral physical examination. Dysphagia was evaluated using modified barium swallow studies (MBSS) interpreted by a speech-language pathologist. Swallowing outcomes were dichotomized as (1) safe and effective swallowing (with or without formal MBSS evaluation) and (2) mild-to-moderate dysphagia per MBSS [14]. Follow-up duration for each outcome was recorded, and mean values were calculated.

Patient demographics (age, gender, smoking status), clinical variables (comorbidities, prior or post radiation and chemotherapy, diagnosis, tumor stage, and tumor location), peri- and postoperative variables (surgical defect area, graft size, graft thickness, suture type, bolster placement, pie-crust status of the graft, postoperative diet, and postoperative antibiotics) were recorded. Smoking status was categorized as either never smoked or any history of smoking, including both former and current use. T-stage was determined from the final surgical pathology report according to the American Joint Committee on Cancer (AJCC) 8th Edition Tumor, Node, Metastasis (TNM) classification [15]. Bolster placement refers to securing the graft to the wound bed using a tie-over dressing to maintain graft-bed contact and minimize shearing forces. Pie-crusting refers to fenestration of the graft with small perforations to allow fluid egress and improve conformity to the wound bed. Postoperative antibiotic use was recorded as a binary variable (yes vs no). Due to variability in antibiotic selection and duration across patients, specific regimens and duration of therapy were not analyzed. 

The University of Arizona Institutional Review Board approved this study (IRB # STUDY00002741) on October 7, 2025. Given the retrospective nature of the study, the requirement for informed consent was waived by the Institutional Review Board.

Statistical analysis was conducted using Statistical Product and Service Solutions (SPSS, version 31; IBM SPSS Statistics for Windows, Armonk, NY). Given the retrospective nature of the study, no a priori sample size calculation was performed. Due to the small sample size and non-normal distribution of continuous variables, nonparametric tests were used. Categorical variables were compared using Fisher's exact test due to small cell counts. Continuous variables were analyzed using the Mann-Whitney U test. Statistical significance was defined as p ≤ 0.05. Suture type and postoperative diet were reported descriptively due to limited variability in suture selection and large variation in dietary protocols, which prevented meaningful statistical analysis.

## Results

Cohort demographics and comorbidities

A total of 39 patients were included in this study. The mean age was 63.9 years (SD = 13.2), and 23 were male (59%). Mean follow-up time was 1.66 years (SD = 1.54). Eleven patients (28.2%) had never smoked, while 28 (71.8%) were current or former smokers. Common comorbidities included type II diabetes mellitus (12.8%), hypertension (10.3%), and alcohol use disorder (10.3%). Due to small subgroup sizes, chemotherapy and radiation were collapsed into binary categories: any (prior or post) versus none. Seven (17.9%) received chemotherapy (with or without radiation), and 13 (33.3%) received radiation. These groups were not mutually exclusive. Thirty-six (92.3%) had invasive squamous cell carcinoma, one (2.6%) had head and neck sarcoma, one (2.6%) had carcinoma in situ, and one (2.6%) had hyperkeratosis. T-stage was dichotomized into early (T1-T2) and late stage (T3-T4) for analysis. Early T-stage tumors were present in 28 patients (71.8%), and late-stage tumors in 10 patients (25.6%). One patient (2.6%) had a benign lesion and was excluded from the statistical analysis of T-stage. Lesion location was grouped as either tongue or buccal mucosa/FOM for statistical analysis. A total of 31 lesions (79.5%) were located on the tongue, while eight lesions (20.5%) involved the buccal mucosa or FOM. Refer to Table 1 for cohort demographics and clinical variables.

**Table 1:** Cohort demographics and clinical and operative variables NPO: Nil per os

Variable	Total cohort (n = 39)
Age (Years), Mean (SD)	63.9 (13.15)
Follow-Up Time (Years), Mean (SD)	1.66 (1.54)
Gender	
Male	23 (59.0%)
Female	16 (41.0%)
Smoking Status	
Never	11 (28.2%)
Former	8 (20.5%)
Light (1-20 Pack-Years, or Cigar Use)	11 (28.2%)
Heavy (>20 Pack-Years)	9 (23.1%)
Comorbidities	
Type II Diabetes Mellitus	5 (12.8%)
Hypertension	4 (10.3%)
Alcohol Use Disorder	4 (10.3%)
Radiation or Chemotherapy Exposure	
Chemotherapy Exposure	7 (17.9%)
Radiation Exposure	13 (33.3%)
Diagnosis	
Invasive Squamous Cell Carcinoma	36 (92.3%)
Sarcoma of the Head and Neck	1 (2.6%)
Carcinoma in Situ	1 (2.6%)
Hyperkeratosis	1 (2.6%)
T-stage	
T1	12 (30.8%)
T2	16 (41.0%)
T3	8 (20.5%)
T4	2 (5.1%)
Benign	1 (2.6%)
Lesion Location	
Tongue	31 (79.5%)
Buccal Mucosa or Floor of Mouth	8 (20.5%)
Surgical Defect Area (cm²), Mean (SD)	11.13 (4.24)
Graft Size (cm²), Mean (SD)	16.14 (13.85)
Graft Thickness (in), Mean (SD)	0.016 (0.002)
Suture Type	
3-0 Chromic	37 (94.9%)
3-0 Vicryl	1 (2.6%)
4-0 Chromic	1 (2.6%)
Intraoperative Variables	
Bolster Placed	6 (15.4%)
Pie-Crusted Graft	10 (25.6%)
Postoperative Diet	
NPO 72h → Clears → Full Liquids	11 (28.2%)
NPO 48h → Clears → Full Liquids	7 (17.9%)
Clear Liquids 6d → Soft Diet	7 (17.9%)
Full Liquid Diet	9 (23.1%)
Regular Diet	1 (2.6%)
No Diet Recorded	4 (10.3%)
Postoperative Antibiotics	16 (41.0%)

Clinical variables

The average surgical defect area was 11.13 cm² (SD = 4.24), with an average graft size of 16.14 cm² (SD = 13.85) and graft thickness of 0.016 in (SD = 0.002). The most common suture type was 3-0 chromic in 37 patients (94.9%), followed by 3-0 Vicryl in one patient (2.6%), and 4-0 chromic in one patient (2.6%). Intraoperatively, six patients (15.4%) had a bolster placed, and 10 (25.6%) had the skin graft pie-crusted. Postoperative diet varied widely, ranging from prolonged nil per os (NPO) status to immediate advancement to regular diet. Eleven patients (28.2%) were NPO for 72 hours followed by clear liquids and then full liquids, seven (17.9%) were NPO for 48 hours followed by clear liquids and then full liquids, seven (17.9%) were on a clear liquid diet for six days followed by a soft diet, nine (23.1%) were on a full liquid diet, and one (2.6%) was on a regular diet. Diet was not reported in four patients (10.3%). Sixteen patients (41.0%) received postoperative antibiotics. Refer to Table 1 for operative variables.

Predictors of graft success

Out of 39 patients, seven had successful skin graft take. Bolster placement (p = 0.059) and pie-crusted grafts (p = 0.057) trended toward significance for association with skin graft success. Demographic and clinical factors, including male gender (p = 1.0), smoking history (p = 0.379), type II diabetes mellitus (p = 0.563), hypertension (p = 1.0), alcohol use disorder (p = 1.0), prior or post-chemotherapy (p = 1.0) and radiation (p = 0.666), late T-stage (p = 0.65), and tumor location (tongue vs other, p = 0.137), did not significantly affect skin graft take. Operative variables, including surgical defect area (p = 0.730), graft size (p = 0.402), skin graft thickness (p = 0.299), and postoperative antibiotics (p = 0.415), did not significantly affect skin graft take. Due to limited variability and small sample size, suture type and postoperative diet were reported descriptively. Refer to Table 2 for categorical variables, and Table 3 for continuous variables.

**Table 2:** Categorical variables grouped by skin graft success Data are presented as n (%). P-values were calculated using two-sided Fisher's exact tests comparing successful versus failed skin graft take.

Variable	Successful skin graft take (n=7)	Failed skin graft (n=32)	P-value (2-sided)
Male Gender	4 (17.4%)	19 (82.6%)	1.0
Female Gender	3 (18.8%)	13 (81.3%)	1.0
Smoking History	4 (14.3%)	24 (85.7%)	0.379
Type II Diabetes Mellitus	0 (0%)	5 (100%)	0.563
Hypertension	0 (0%)	4 (100%)	1.0
Alcohol Use Disorder	0 (0%)	4 (100%)	1.0
Prior or Post Chemo	1 (14.3%)	6 (85.7%)	1.0
Prior or Post Radiation	3 (23.1%)	10 (76.9%)	0.666
Late Cancer Stage (T3/T4)	1 (10%)	9 (90%)	0.65
Tongue Tumor Location	4 (12.9%)	27 (87.1%)	0.137
Pie-Crusted Skin Graft	4 (40%)	6 (60%)	0.057
Bolster Placement	3 (50%)	3 (50%)	0.059
Postoperative Antibiotics	4 (25%)	12 (75%)	0.415

**Table 3:** Continuous variables grouped by skin graft success Data are presented as mean ± standard deviation. P-values represent two-sided Mann-Whitney U tests comparing successful versus failed skin graft take.

Variable	Successful skin graft take (n=7)	Failed skin graft (n=32)	P-value (2-sided)
Age (Years)	65.7 (17.2)	63.07 (12.6)	0.654
Surgical Defect Area (cm^2^)	11.5 (4.7)	10.9 (4.3)	0.730
Graft Size (cm^2^)	24.9 (27.5)	14.1 (7.7)	0.402
Skin Graft Thickness (in)	0.017 (0.002)	0.015 (0.002)	0.299

Functional complications following reconstruction

Tongue tethering was observed in seven patients (17.9%), and dysphagia in six patients (15.4%). Among patients with dysphagia, the mean time from surgery to speech-language pathology (SLP) evaluation was 3.5 months (SD = 4.3). No clinical or operative variables were significantly associated with tongue tethering. However, patients who received chemotherapy (p = 0.006) or radiation (p = 0.011) were more likely to experience mild-to-moderate dysphagia. Functional outcomes are summarized in Tables 4-7.

**Table 4:** Categorical variables grouped by dysphagia Data are presented as n (%). P-values were calculated using two-sided Fisher's exact tests comparing safe and effective swallowing versus mild-to-moderate dysphagia.

Variable	Safe and effective swallowing (n=33)	Mild-to-moderate dysphagia (n=6)	P-value (2-sided)
Male Gender	20 (86.9%)	3 (13%)	0.674
Female Gender	13 (81.3%)	3 (18.8%)	0.674
Smoking History (+)	25 (89.2%)	3 (10.7%)	0.323
Type II Diabetes Mellitus	4 (80%)	1 (20%)	1.0
Hypertension	4 (100%)	0 (0%)	1.0
Alcohol Use Disorder	3 (75%)	1 (25%)	0.502
Prior or Post Chemo	3 (42.9%)	4 (57.1%)	0.006
Prior or Post Radiation	8 (61.5%)	5 (38.5%)	0.011
Late T-Stage (T3/T4)	8 (80%)	2 (20%)	0.644
Tongue Tumor Location	25 (80.6%)	6 (19.4%)	0.313
Pie-Crusted Skin Graft	7 (70%)	3 (30%)	0.163
Bolster Placement	5 (83.3%)	1 (16.7%)	1.0
Postoperative Antibiotics	13 (81.3%)	3 (18.8%)	0.674

**Table 5:** Continuous variables grouped by dysphagia Data are presented as mean ± standard deviation. P-values were calculated using two-sided Mann-Whitney U tests comparing safe and effective swallowing versus mild-to-moderate dysphagia.

Variable	Safe and effective swallowing (n=33)	Mild-to-moderate dysphagia (n=6)	P-value (2-sided)
Age (Years)	64.70 (13.18)	57.79 (14.0)	0.198
Surgical Defect Area (cm^2^)	10.55 (4.38)	13.52 (2.8)	0.182
Graft Size (cm^2^)	15.97 (14.94)	17.05 (6.30)	0.343
Skin Graft Thickness (in)	0.016 (0.002)	0.016 (0.002)	0.894

**Table 6:** Categorical variables grouped by tongue tethering Data are presented as n (%). P-values were calculated using two-sided Fisher's exact tests comparing non-tethered versus tethered patients.

Variable	Not tethered (n=32)	Tethered (n=7)	P-value (2-sided)
Male Gender	20 (86.9%)	3 (13%)	0.415
Female Gender	12 (75%)	4 (25%)	0.415
Smoking History	24 (85.7%)	4 (14.3%)	0.379
Type II Diabetes Mellitus	4 (80%)	1 (20%)	1.0
Hypertension	4 (100%)	0 (0%)	1.0
Alcohol Use Disorder	3 (75%)	1 (25%)	0.563
Prior or Post Chemo	5 (71.4%)	2 (28.6%)	0.588
Prior or Post Radiation	9 (69.2%)	4 (30.8%)	0.194
Late T- Stage (T3/T4)	8 (80%)	2 (20%)	1.0
Tongue Tumor Location	26 (83.9%)	5 (16.1%)	0.617
Pie-Crusted Skin Graft	7 (70%)	3 (30%)	0.344
Bolster Placement	3 (50%)	3 (50%)	0.059
Postoperative Antibiotics	13 (81.3%)	3 (18.8%)	1.0

**Table 7:** Continuous variables grouped by tongue tethering Data are presented as mean ± standard deviation. P-values were calculated using two-sided Mann-Whitney U tests comparing non-tethered versus tethered patients.

Variable	Not tethered (n=32)	Tethered (n=7)	P-value (2-sided)
Age (Years)	63.18 (12.88)	65.24 (16.31)	0.554
Surgical Defect Area (cm^2^)	10.85 (4.41)	11.81 (3.82)	0.703
Graft Size (cm^2^)	15.83 (15.25)	17.47 (4.86)	0.212
Skin Graft Thickness (in)	0.016 (0.002)	0.017 (0.002)	0.299

## Discussion

Surgical resection is the standard of care for malignant, premalignant, benign, and inflammatory oral cavity lesions [3,6,16]. While resection offers significant mortality benefit, it results in large intraoral defects that impair speech, mastication, and swallowing [4,7,17-19]. For defects that are too extensive to close by primary closure or heal by secondary intent, STSGs are commonly used to resurface the wound bed and restore function without adding excessive bulk [7]. The oral cavity presents a uniquely hostile environment for graft survival due to constant mechanical stress from mastication and speech, making complete graft take particularly challenging [12,13,20,21]. Predictors of intraoral STSG success remain poorly defined. To our knowledge, this is the first study to analyze demographic, clinical, and operative predictors of intraoral STSG survival.

In this retrospective series of 39 patients undergoing intraoral STSG after resection, we observed an overall complete graft take rate of 17.9%, with bolster placement and pie-crusting showing trends toward improved graft survival. Demographic factors, comorbidities, and tumor characteristics were not significantly associated with graft survival. Similarly, operative and postoperative variables, including wound size, graft size, graft thickness, and postoperative antibiotic use, were not associated with outcomes.

Graft survival depends on early adherence and revascularization, through the process of imbibition, inosculation, and neovascularization [22]. Disruptions in this delicate process, such as shear forces, hematoma, infection, or poor perfusion, can lead to graft failure [22]. Histologic studies have further demonstrated that intraoral STSGs maintain epidermal architecture and remain viable for years, even after adjuvant radiation, although they do not transform into mucosa [23].

Tie-over bolsters, which consist of a combination of ointment, gauze or xeroform, and cotton secured using tie-over sutures, are traditionally used to immobilize grafts, reduce shearing forces, and maintain close graft-bed contact. Schramm et al. first described tie-over bolster use in the oral cavity, reporting a 97% take rate in 57 patients, though many required temporary tracheostomy for airway protection [24]. In a prospective study of early-stage FOM cancers reconstructed with STSGs, Larson et al. reported excellent outcomes, with no complete graft losses among 24 patients treated with STSG and xeroform bolsters (removed at five to seven days) [20]. By contrast, Patel et al. conducted a systematic review across multiple anatomic sites and found no consistent improvement in graft take with bolsters compared to alternatives such as quilting, peripheral sutures, or foam dressings. Notably, none of the included studies specifically examined the oral cavity [13]. Despite the established role of bolsters in general graft fixation, no prior study has evaluated clinical predictors of graft success.

In our cohort, bolster placement and pie-crusting were the only operative factors that trended toward improved long-term graft survival, with 50% of bolstered grafts achieving complete epithelialization at final follow-up compared to 12.5% without a bolster. Of note, the three patients with successful bolstered grafts were located on the buccal mucosa or FOM, whereas none of the three bolstered grafts placed on the tongue survived. While limited by the small number of patients, this observation raises the possibility that anatomic site may influence the effectiveness of bolster fixation. The increased tongue mobility and associated shear forces, compared to the relatively immobile buccal mucosa and FOM, likely account for this anatomic difference. Unlike Schramm et al., who assessed short-term healing, our definition of graft success required continued skin graft viability at the latest clinic visit [24]. The mean follow-up in our series exceeded one and a half years, allowing assessment of long-term graft survival. Larson et al. similarly reported no complete graft losses, with a mean follow-up of 41 months, though their primary focus was patient-reported functional outcomes rather than graft take [20]. Our stricter evaluation of graft take likely explains our lower absolute success rate but strengthens the relevance of our observed trends. While our findings differ from Patel et al.'s conclusions, the difference may reflect the unique challenges of intraoral grafting [13]. Nevertheless, our small sample warrants cautious interpretation.

Pie-crusting, or fenestration of STSGs, is often performed to facilitate drainage and improve graft conformity [22]. Although evidence in the oral cavity is limited, a large retrospective review by Shin et al. involving more than 300 lower-extremity STSGs demonstrated higher graft failure rates when fenestration was not performed [25]. Although we observed a non-significant trend toward improved take with pie-crusting, the effect did not reach significance, potentially due to the hostile intraoral environment or limited sample size. Other operative variables, including wound size, graft size, graft thickness, and postoperative antibiotics, were not associated with graft outcomes. Collectively, these results suggest that bolster placement and pie-crusting may contribute to improved graft survival, though further investigation in larger, prospective studies is necessary.

Comorbidities such as smoking, diabetes, and alcohol use are well-established risk factors for impaired wound healing and postoperative complications in head and neck surgery [26,27]. The evidence regarding radiation therapy for STSG, however, is mixed. Although radiation has been associated with higher complication and failure rates in free flap reconstruction, its effect on STSG success appears less pronounced [28]. Petruzzelli et al. demonstrated no significant histologic differences between radiated and non-radiated intraoral STSGs, while Bui et al. reported that, when radiation was initiated approximately eight weeks after STSG placement, only 10% of grafts experienced partial or complete failure [23,29]. Taken together, these findings support our observation that radiation did not significantly impact graft survival in our cohort. Although it should be noted that radiation and chemotherapy exposure were analyzed as a combined variable for pre- and postoperative therapy, given the limited subgroup size. Additionally, T-stage, while not directly linked to graft take, may indirectly affect outcomes by necessitating larger or deeper resections with compromised vascularity. However, in our series, late T-stage disease was not associated with graft failure. Similarly, tumor location on the tongue had no significant influence on graft take. These findings suggest that demographic and tumor-related factors may be less critical to intraoral STSG outcomes compared to technical aspects such as secure fixation.

Tongue tethering and dysphagia are recognized functional risks of intraoral STSG reconstruction. In our series, seven (17.9%) patients developed tethering, and six (15.4%) patients experienced dysphagia. Larson et al. reported higher rates of dysfunction, with 42% of patients experiencing difficulty swallowing solid foods after FOM STSG. However, their results were based on patient-reported questionnaires rather than instrumental swallow studies [20]. In our cohort, tethering was not associated with clinical variables, but both chemotherapy and radiation were significantly associated with dysphagia. This contrasts with Larson et al., who found no significant link between adjuvant radiation and swallowing outcomes [20]. Given our small sample size and the consolidation of pre- and postoperative treatments within each variable, these findings should be interpreted cautiously, though they are consistent with prior literature linking radiation to dysphagia [30,31].

These findings have several clinical implications. The observed trend toward improved graft success with bolster placement and pie-crusting suggests that optimizing graft fixation and graft-bed contact may play an important role in STSG success. Given the lack of association between demographic and tumor-related factors and graft success, the graft fixation technique may play a larger role in outcomes than patient or tumor characteristics. Additionally, the association between dysphagia and adjuvant chemotherapy and radiation highlights the importance of early multidisciplinary care for these patients. While these findings should be interpreted in the setting of a small retrospective case series, they may inform surgical decision-making and future prospective studies.

This study has several limitations. Its retrospective design and single institution setting limit generalizability, and the relatively small sample size may have reduced statistical power to detect associations between clinical or operative variables and graft outcomes. Graft take was determined retrospectively from physical examination documentation, introducing potential variability in reporting and assessment, which may partly explain the relatively low number of grafts classified as successful compared to prior literature.

Despite these limitations, this study has several strengths. This paper evaluates demographic and clinical predictors of intraoral STSG survival in patients who did not undergo free flap reconstruction. By analyzing a well-characterized cohort and a broad range of operative and patient factors, this work provides novel insight into predictors of graft success within the uniquely challenging anatomical environment of the oral cavity.

## Conclusions

In this retrospective analysis of intraoral STSG take, bolster placement, and pie-crusting demonstrated trends toward improved long-term graft survival. Broader demographic, clinical, and tumor-related variables were not predictive of graft take, suggesting that technical factors such as secure fixation may play a greater role in optimizing outcomes. Functional complications, including tongue tethering and dysphagia, were uncommon, though dysphagia was significantly associated with patients who received radiation or chemotherapy. Larger, prospective studies are needed to better define predictors of intraoral split-thickness skin graft success and optimize outcomes in oral cavity reconstruction.
